# IGF2BP2 Overexpression Predicts Poor Prognosis and Correlates with PD-L1 Expression in Intrahepatic Cholangiocarcinoma

**DOI:** 10.3390/biomedicines14040929

**Published:** 2026-04-19

**Authors:** Jianan Shen, Aihua Yang, Xintao He, Tianyi Dai, Zexuan Hui, Youxiang Ding, Li Zhao, Jun Chen

**Affiliations:** 1 Department of Pathology, Nanjing Drum Tower Hospital, School of Basic Medicine and Clinical Pharmacy, China Pharmaceutical University, Nanjing 210000, China; jia_nan_shen@163.com; 2 Public Experimental Platform, China Pharmaceutical University, Nanjing 211198, China; 3 Department of Pathology, Nanjing Drum Tower Hospital, Clinical College of Nanjing University of Chinese Medicine, Nanjing 210000, China; yang1320219216@163.com (A.Y.); twist51542@gmail.com (X.H.); daitianyi202202@163.com (T.D.); huizexuan1013@126.com (Z.H.)

**Keywords:** intrahepatic cholangiocarcinoma, IGF2BP2, prognosis, immune checkpoint, tumor microenvironment

## Abstract

**Background**: The immunologically cold nature and immunosuppressive tumor microenvironment (TME) of intrahepatic cholangiocarcinoma (ICC) contribute to its poor prognosis. This study aims to identify novel biomarkers related to prognosis and TME in ICC. **Methods**: We first identified the high expression of m6A reader insulin-like growth factor 2 mRNA binding protein 2 (IGF2BP2) in ICC through bioinformatics screening. Subsequently, a retrospective study was conducted on 224 ICC patients who had undergone radical resection. The expression levels of IGF2BP2 and programmed death ligand 1 (PD-L1) were detected in a tissue microarray (TMA) using immunohistochemistry (IHC). The co-localization of IGF2BP2, PD-L1, programmed cell death protein 1 (PD-1), and CD8^+^T cells was evaluated by multiple immunofluorescence techniques. **Results**: IHC confirmed a significant upregulation of IGF2BP2 in tumor tissues compared with normal bile duct epithelia (*p* < 0.05). IGF2BP2 expression was positively correlated with PD-L1 expression (TPS *R* = 0.215, *p* = 0.016; CPS *R* = 0.295, *p* = 0.008). High IGF2BP2 expression was associated with increased PD-L1/PD-1 positivity and reduced CD8^+^T cell infiltration. Kaplan–Meier analysis revealed significantly worse 3-year overall survival (OS: 20.56% vs. 29.91%, *p* = 0.0291) and recurrence-free survival (RFS: 9.72% vs. 18.56%, *p* = 0.0372) in the IGF2BP2-high group. Multivariate analysis identified IGF2BP2 as an independent risk factor for both OS (HR = 1.683, *p* = 0.044) and RFS (HR = 1.946, *p* = 0.042). **Conclusions**: IGF2BP2, as a potential biomarker and independent prognostic factor for ICC, is associated with increased PD-L1 expression.

## 1. Introduction

Primary liver cancer is a leading cause of cancer-related mortality worldwide [1]. Intrahepatic cholangiocarcinoma (ICC) is the second most common primary liver malignancy after hepatocellular carcinoma [2], accounting for approximately 15% of all primary liver cancers. Its incidence has increased in recent years [3]. Optimal pharmacologic treatment strategies remain ill-defined and lack standardization [4]. Surgery remains the cornerstone of curative management [5].

N^6^-methyladenosine (m^6^A) is a dynamic, reversible RNA modification regulated by writers, erasers, and readers, which governs post-transcriptional gene regulation in eukaryotes [6]. Among the m^6^A readers, the insulin-like growth factor 2 mRNA binding protein (IGF2BP) family stabilizes coding/non-coding RNAs to modulate RNA metabolism [7]. Specifically, IGF2BP2 functions as a multifunctional oncogenic regulator that binds to various RNA species (mRNAs, miRNAs, lncRNAs, circRNAs) and promotes tumorigenesis via aberrant post-transcriptional regulation [8,9]. Previous studies based on TCGA or GEO databases have suggested the potential of IGF2BP2 as a pan-cancer prognostic biomarker [10]. However, its functional landscape in ICC remains to be elucidated. Nevertheless, bioinformatics analysis primarily serves as an auxiliary data-processing tool and cannot accurately predict the precise role of a given gene in specific tumor types. Therefore, its clinical significance warrants further validation through expanded patient cohorts.

Immune evasion—a hallmark of cancer according to Hanahan-Weinberg—drives pathogenesis in ICC [11,12], an archetypal immune-cold tumor with an immunosuppressive microenvironment that confers therapeutic resistance [13]. Clinically, elevated PD-L1 expression is associated with aggressive features and poor survival [14]. In contrast, T-cell infiltrates exhibit divergent prognostic implications: CD8+/CD4+ T-cell infiltration is correlated with improved survival and reduced invasiveness, whereas enrichment of FoxP3+ regulatory T cells (Tregs) predicts adverse outcomes [15]. Furthermore, our previous research indicates that CD4 and FoxP3 are often used as combined markers in immunotherapy [16]. Across malignancies, IGF2BP2 shapes an immunosuppressive tumor microenvironment by post-transcriptionally stabilizing target transcripts in an m6A-dependent manner. In pancreatic ductal adenocarcinoma, it stabilizes oncogenic transcripts and promotes M2-type tumor-associated macrophage polarization [17]. In melanoma, IGF2BP2 ablation enhances MHC class I antigen presentation and CD8+ T-cell interferon-γ production [18]. In colorectal cancer (CRC) and gastric cancer (GC), IGF2BP2 directly binds to and stabilizes programmed death-ligand 1 (PD-L1) mRNA via KH-domain-mediated RNA recognition, sustaining PD-L1 overexpression and attenuating CD8+ T-cell cytotoxicity through PD-1/PD-L1 axis activation [19,20]. However, its role in ICC remains unclear. The clinical significance and immunomodulatory functions of IGF2BP2 in ICC remain unexplored, with a lack of histopathologic validation and comprehensive immune-landscape correlation. Therefore, this study aims to investigate the significance of IGF2BP2 expression in distinct molecular and histological subtypes of ICC and explore its potential association with immune evasion based on inferences drawn from clinical observations.

## 2. Materials and Methods

### 2.1. Bioinformatic Analysis

Transcriptomic datasets GSE107943 and GSE119336 were retrieved from the Gene Expression Omnibus (GEO), https://www.ncbi.nlm.nih.gov/geo/ (accessed on 9 April 2026), and differential expression analysis was conducted using GEO2R to generate volcano plots of m6A-related differentially expressed genes. Pan-cancer transcriptomic data were obtained from The Cancer Genome Atlas (TCGA). Gene-level heat maps and tumor–normal differential expression were derived via GEPIA, http://gepia.cancer-pku.cn/detail.php (accessed on 9 April 2026). For the IGF2BP family, the tumor vs. normal expression within the GEO datasets was visualized as dot plots. In TCGA-CHOL, correlations between IGF2BP2 and immune checkpoint genes were queried in GEPIA. Meanwhile, associations between IGF2BP2 and immunomodulators (inhibitors/stimulators/MHC molecules) and lymphocyte infiltration were analyzed using TISIDB, http://cis.hku.hk/TISIDB/ (accessed on 9 April 2026), and interactive pan-cancer heatmaps were generated to display the relationships.

### 2.2. Patient Cohort and Data Collection

**Figure 1:**
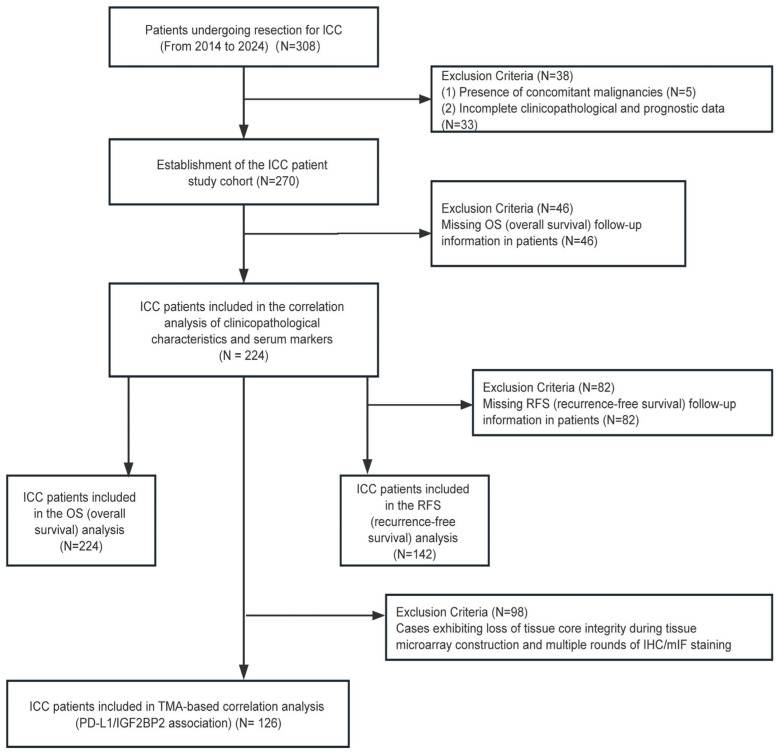
Inclusion and Exclusion Flowchart for ICC Patients.

This study was approved by the Medical Ethics Committee of Nanjing Drum Tower Hospital, affiliated with Nanjing University Medical School (Approval No. 2013-081-12). Clinicopathological and prognostic data of the enrolled ICC patients were collected, and inclusion and exclusion criteria were established (Figure 1).

The inclusion criteria were as follows:

1. Patients with no residual tumor present after curative surgical resection.
2. Histologically confirmed intrahepatic cholangiocarcinoma by the Department of Pathology at our hospital.
3. Complete clinicopathological data available for collection.
4. Complete follow-up data available for collection.
5. Written informed consent obtained from patients and their families for the use of tissue samples.

The exclusion criteria were:

1. Presence of other concurrent malignancies, such as intrahepatic cholangiocarcinoma combined with hepatocellular carcinoma, metastatic liver cancer, extrahepatic cholangiocarcinoma, or gallbladder carcinoma.
2. Receipt of neoadjuvant therapy prior to surgery.
3. Incomplete clinicopathological or prognostic data.

A retrospective cohort of 224 histopathologically confirmed ICC patients who underwent surgical resection was enrolled. Paired tumor and adjacent non-tumorous tissues (*n* = 224) were sectioned at 2 μm. Clinicopathological parameters, serum biomarkers, and survival outcomes were collected. Follow-up was conducted through telephone surveys until May 2024.

### 2.3. Tissue Microarray Construction

Representative tumor regions (≥1.5-mm diameter) were annotated on hematoxylin and eosin (H&E) sections and cored from donor formalin-fixed, paraffin-embedded (FFPE) blocks using 1.5-mm needles. Duplicate cores per case were arrayed into a recipient block. Core integrity was verified by H&E staining, and detached or mispositioned samples were excluded during quality control.

After these steps, a total of 126 cases were fully included in the final cohort. The reduction from the 224-patient cohort to the 126-case TMA sub-cohort was due to technical issues, such as core detachment during multi-round staining.

### 2.4. Immunohistochemistry

Formalin-fixed paraffin sections were dewaxed, rehydrated, and subjected to EDTA-based antigen retrieval. After peroxidase blocking, slides were incubated overnight at 4 °C with anti-IGF2BP2 (Proteintech, Chicago, IL, USA, 11601-1-AP, 1:800) or anti-PD-L1 SP142 (Roche, Basel, Switzerland, 1:100), followed by an HRP-polymer secondary antibody (30 min, room temperature). Signals were developed with DAB (Zhongshan Golden Bridge) and counterstained with hematoxylin. Every IHC run included external positive and negative controls to ensure staining fidelity.

The staining intensity of IGF2BP2 was independently assessed by two senior pathologists in a blinded manner. For immunohistochemistry (IHC), we employed the IHC-score system, which is calculated by multiplying the staining intensity score (0–3) by the percentage of positive cells (0–100%). Using the median IHC-score as the cutoff point, we classified IGF2BP2 expression into high and low groups.

PD-L1 expression (clone SP142) was evaluated by two pathologists blinded to clinical data, who scored partial or complete membranous staining on tumor cells, as well as membranous and cytoplasmic staining on immune cells. Discrepancies between the observers were resolved by consensus. PD-L1 was scored using the Tumor Proportion Score (TPS = [PD-L1^+^ tumor cells with membrane staining/total viable tumor cells] × 100%) and the Combined Positive Score (CPS = [PD-L1^+^ tumor cells + PD-L1^+^ immune cells]/total viable tumor cells × 100). PD-L1 scores were treated as continuous variables to maximize the sensitivity of correlation analysis with IGF2BP2 expression.

### 2.5. Multiplex Immunofluorescence

Using the Opal™ 7-Color Automation Kit (Akoya Biosciences, Marlborough, MA, USA), multiplex staining was carried out with fluorophore-conjugated antibodies: IGF2BP2 (Proteintech 11601-1-AP/Opal 570), PD-1 (Abcam ab137132/Opal 690, Cambridge, UK), PD-L1 (Roche SP142/Opal 620), and CD8 (Abcam ab237709/Opal 480), along with DAPI nuclear counterstain. Following deparaffinization and antigen retrieval, sequential staining cycles were carried out. For multiplex immunofluorescence, quantitative multispectral analysis was performed using the Vectra Polaris™ system, allowing for the precise determination of cell densities and protein co-localization within representative tumor regions.

### 2.6. Statistical Analysis

R 4.0 and GraphPad Prism (v8.0) were used for analyses. IGF2BP2–clinicopathological associations were examined by χ^2^ or Fisher’s exact tests. Survival was evaluated with Kaplan–Meier curves and log-rank tests. Factors significantly associated with survival in univariate analysis (*p* < 0.05) were included in a multivariate Cox proportional hazards model using the “Enter” method to estimate independent hazard ratios (HRs) and 95% confidence intervals (CIs). This approach ensured that established clinical prognosticators remained in the model to minimize selection bias.

In GEO2R, differentially expressed genes were defined by |log_2_ FC| ≥ 1 and Benjamini–Hochberg FDR-adjusted *p* < 0.05. For IGF2BP family genes in GEO datasets, tumor–normal differences were tested using two-sided Student’s *t*-tests and visualized as dot plots. Genes significantly correlated with IGF2BP2 underwent GO/KEGG enrichment via cluster Profiler (*p* < 0.05; BH-adjusted *q* < 0.05).

For bioinformatic screening in the TCGA-CHOL dataset, a correlation threshold of |*R*| > 0.3 and *p* < 0.05 was applied to identify robust immune correlates. For IHC validation in our clinical cohort, statistical significance was defined as *p* < 0.05. TISIDB and pan-cancer analyses likewise applied Spearman correlations to generate heatmap matrices.

Unless otherwise specified, all tests were two-sided, and multiple comparisons were controlled by the Benjamini–Hochberg procedure. A two-tailed *p* < 0.05 was considered statistically significant.

## 3. Results

### 3.1. IGF2BP2 Is a Differentially Expressed m6A-Related Gene Associated with the Immune Microenvironment in ICC

**Figure 2:**
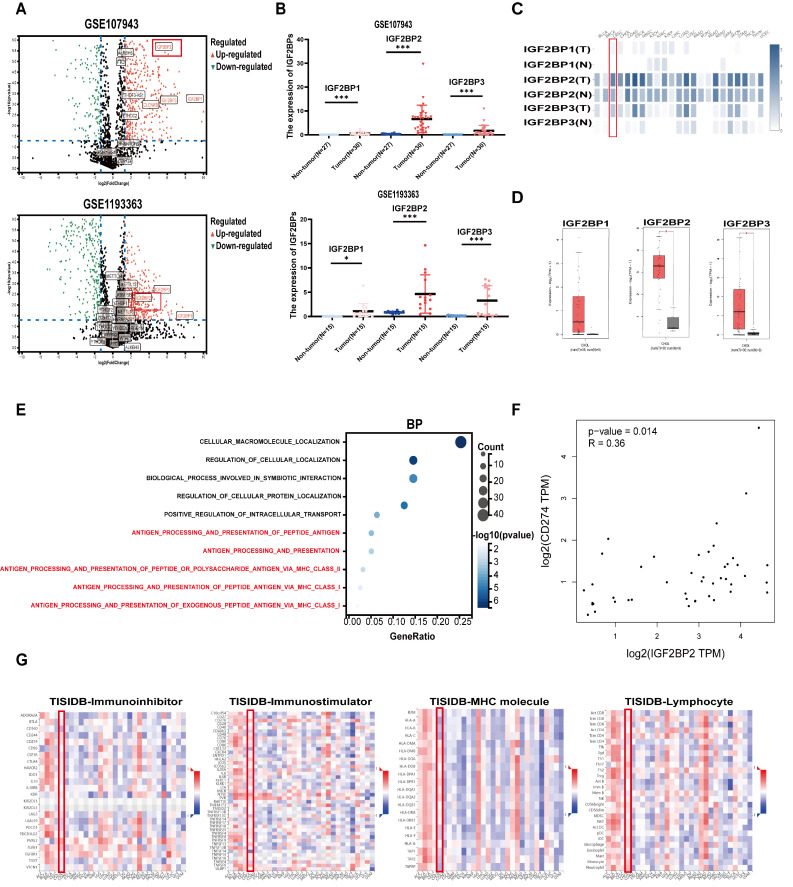
Bioinformatic Analysis and Tumor-Immune Interaction Analysis for IGF2BP2. (**A**) Volcano plots of m6A regulators in ICC (GSE107943/GSE119336). (**B**) IGF2BP differential expression in GSE107943/GSE119336 (* *p* < 0.05, *** *p* < 0.001). (**C**) IGF2BP expression across tumors vs. normal. (**D**) IGF2BPs in CHOL tumors vs. normal (* *p* < 0.05). (**E**) GO enrichment (BP) of IGF2BP2-associated genes. (**F**) IGF2BP2 correlation with immune checkpoints (CD274). (**G**) IGF2BP2 expression correlates with immune inhibitor molecules, immune stimulator molecules, MHC molecules, and lymphocyte subsets in CHOL.

Analysis of the GEO datasets (GSE107943/GSE119336) revealed no significant differential expression in m6A writers (e.g., METTL16/METTL14) or erasers (e.g., FTO/ALKBH5) (|log_2_ FC| < 1.5, *p* > 0.05). In contrast, reader genes IGF2BP1/2/3 showed marked tumor upregulation (|log_2_ FC| > 1.5, *p* < 0.01), establishing IGF2BPs as key m6A regulators in ICC (Figure 2A). IGF2BP2 exhibited significantly greater upregulation in ICC tumors compared to normal bile duct tissues (*p* < 0.05) (Figure 2B). TCGA pan-cancer analysis showed significant tumor upregulation of IGF2BP1/2/3, including cholangiocarcinoma (Figure 2C). GEPIA analysis of TCGA-CHOL data revealed that IGF2BP2 expression was significantly elevated in tumors compared to normal tissues (*p* < 0.05), and the upregulation was greater than that of its homologs IGF2BP1/3 (Figure 2D). GO enrichment analyses revealed that genes significantly associated with IGF2BP2 were enriched in immune-related pathways (Figure 2E). GEPIA analysis revealed significant positive correlations (*R* > 0.25, *p* < 0.05) between IGF2BP2 and the immune checkpoint gene CD274 in CHOL (Figure 2F). TISIDB analysis revealed significant Spearman correlations between pan-cancer IGF2BP2 expression and immune inhibitors (e.g., PD-L1, CTLA-4), immune stimulators (e.g., CD276, ENTPD1), MHC molecules, and lymphocytes (e.g., CD8^+^ T cells, Tregs). In cholangiocarcinoma, IGF2BP2 correlated with immunomodulators and tumor-infiltrating lymphocytes (Figure 2G).

### 3.2. High Expression of IGF2BP2 in ICC Is Associated with Malignant Clinicopathological Characteristics, Altered Serum Indices, and Inferior Survival

**Figure 3:**
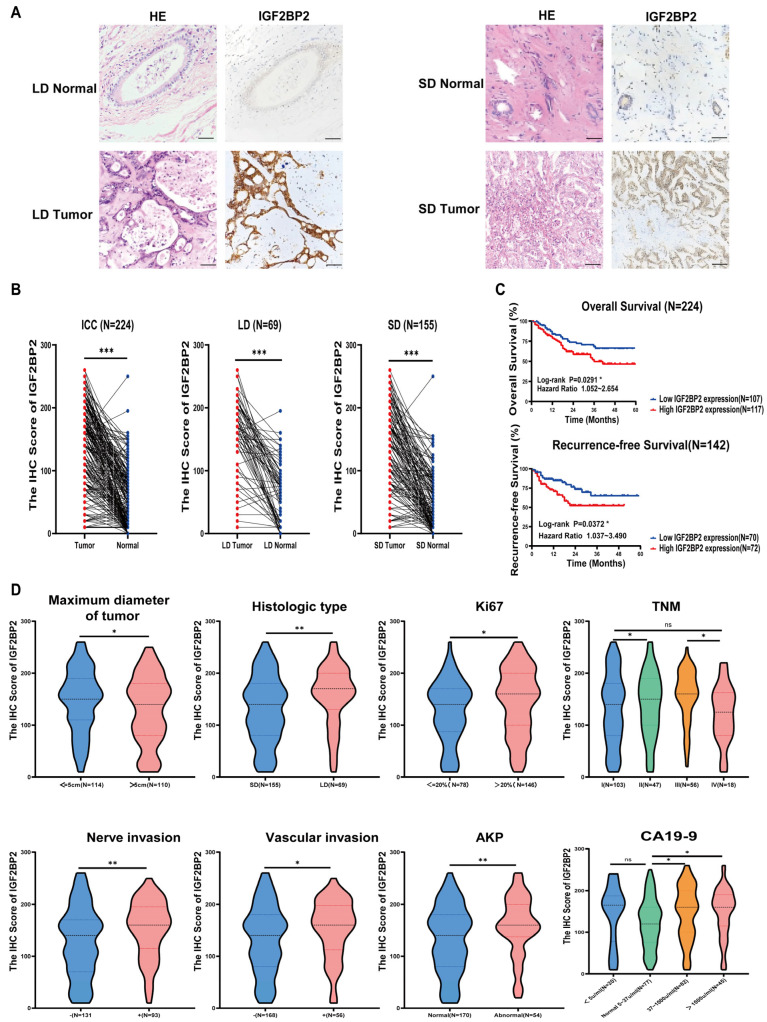
Correlation of IGF2BP2 Expression with Clinicopathological Characteristics, Serum Indices, and Prognosis in ICC. (**A**) HE/IHC staining of normal bile duct vs. ICC tumors (LD/SD; ×200). (**B**) IGF2BP2 IHC in LD/SD types: tumors vs. normals (*** *p* < 0.001). (**C**) Kaplan–Meier OS/RFS curves by IGF2BP2 expression (* *p* < 0.05). (**D**) IGF2BP2 is differentially expressed among maximum diameter of tumor, histologic type, vascular invasio, nerve invasion, TNM (II vs. I, III vs. IV), Ki67, AKP, and CA19-9 (* *p* < 0.05, ** *p* < 0.01).

IGF2BP2 protein is predominantly cytoplasmic in ICC cells, with stronger staining in tumors compared to normal bile ducts. Expression may be higher in the large-duct (LD) type than in the small-duct (SD) type. It is minimal in normal large ducts but virtually absent in normal hepatocytes and small ducts (Figure 3A). IHC validation in 224 ICC specimens revealed that cytoplasmic IGF2BP2 expression was significantly higher in tumor tissues than in paired adjacent bile ducts (*p* < 0.001). This marked increase was consistently observed across both large-duct (*p* < 0.001) and small-duct (*p* < 0.001) subtypes (Figure 3B). Among the 224 ICC cases stratified by IHC, the IGF2BP2-high group (117/224, 52.20%) exhibited significantly worse 3-year outcomes compared to the low-expression group (107/224, 47.80%), with overall survival (OS) dropping from 29.91% to 20.56% (*p* = 0.0291) and recurrence-free survival (RFS) decreasing from 18.56% to 9.72% (*p* = 0.0372) (Figure 3C).

The expression of IGF2BP2 was correlated with the clinicopathological characteristics and serum indices of ICC patients (Table 1). Immunohistochemical analysis revealed that high IGF2BP2 expression was significantly associated with multiple aggressive clinicopathological features and abnormal serum levels in patients with ICC (*p* < 0.05) (Figure 3D). These included histological subtype (LD type) (*p* < 0.001), vascular (*p* = 0.037) and nerve invasion (*p* = 0.011), and advanced TNM stages (particularly showing a notable enrichment trend in Stage III patients) (*p* = 0.030). Furthermore, elevated IGF2BP2 expression levels were significantly correlated with several serological indicators reflecting liver dysfunction and tumor burden, including abnormal serum levels of alanine aminotransferase (ALT) (*p* = 0.021), aspartate aminotransferase (AST) (*p* = 0.016), alkaline phosphatase (AKP) (*p* = 0.015), gamma-glutamyl transpeptidase (GGT) (*p* = 0.029), and carbohydrate antigen 19-9 (CA19-9) (*p* = 0.006).

### 3.3. High Expression of IGF2BP2 Correlated with Upregulated PD-L1/PD-1 Expression and Reduced CD8^+^T-Cell Infiltration in ICC

**Figure 4:**
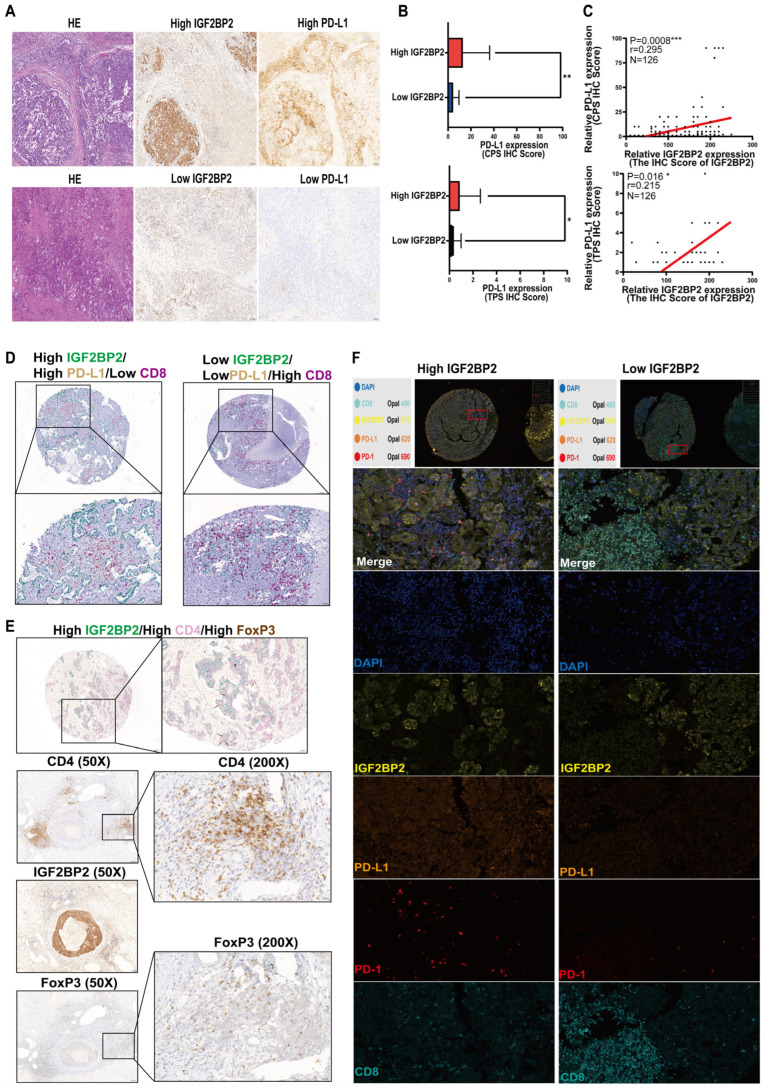
Spatial and Quantitative Association of IGF2BP2 with PD-L1 and Infiltrating CD8+ T Cells in ICC. (**A**) H&E/IGF2BP2/PD-L1 co-staining (×100). (**B**) High IGF2BP2 expression was significantly associated with elevated PD-L1 levels across both scoring modalities, including TPS (* *p* < 0.05) and CPS (** *p* < 0.01). (**C**) Spearman rank correlation analysis revealed that IGF2BP2 expression was positively correlated with PD-L1 levels as measured by both TPS (*r* = 0.215, * *p* < 0.05) and CPS (*r* = 0.295, *** *p* < 0.001), the solid red line denotes the fitted curve derived from Spearman’s rank correlation analysis. (**D**) PD-L1/CD8 distribution in high-IGF2BP2 group vs. low-IGF2BP2 group (×75/×200). (**E**) Co-localization of CD4 and FoxP3 in the high IGF2BP2 expression group (×75/×200), and expression of CD4 and FoxP3 in corresponding pathological sections from the same cases (×50/×100). (**F**) PD-L1/PD-1/CD8 co-localization in high-IGF2BP2 group vs. low-IGF2BP2 group (×30/×200), the area inside the red box represents the field of view after magnification.

Compared with the low IGF2BP2 ICC group, the expression of PD-L1 in tumor and immune cells in the high IGF2BP2 ICC group was increased (Figure 4A). In 126 ICC cases available for TMA analysis, PD-L1 TPS and CPS were significantly higher in the high versus the low IGF2BP2 groups (*p* < 0.05) (Figure 4B). IGF2BP2 expression was positively correlated with PD-L1 TPS (*R* = 0.215, *p* = 0.016) and CPS (*R* = 0.295, *p* = 0.008) (Figure 4C). Multiplex immunohistochemistry revealed that high IGF2BP2 expression is correlated with PD-L1 positivity and reduced CD8^+^T-cell infiltration density, whereas low expression is associated with PD-L1 negativity and elevated CD8^+^T-cell infiltration (Figure 4D). Multiplex immunohistochemistry revealed that high IGF2BP2 expression was accompanied by FoxP3 positivity and CD4^+^ cell infiltration. In corresponding cases exhibiting high IGF2BP2 expression, elevated expression levels of FoxP3 and CD4 immune markers were also observed (Figure 4E). Multiplex immunofluorescence confirmed that high IGF2BP2 expression was correlated with PD-L1^+^/PD-1^+^ phenotypes and reduced CD8^+^ T-cell infiltration density, whereas low expression was associated with PD-L1^−^/PD-1^−^ status and elevated CD8^+^ T-cell infiltration (Figure 4F).

### 3.4. High Expression of IGF2BP2 Was an Independent Prognostic Risk Factor for OS and RFS in ICC Patients

In order to determine the potential of IGF2BP2 as an independent prognostic indicator for ICC, univariate and multivariate analyses were conducted. All variables with a single-factor *p* < 0.05 were incorporated into the multivariate Cox regression model. Multivariate analysis identified IGF2BP2 expression (HR = 1.683, 95% CI = 1.015–2.791; *p* = 0.044), tumor diameter (HR = 2.155, 95% CI = 1.292–3.595; *p* = 0.003), Alb (HR = 2.653, 95% CI = 1.304–5.396; *p* = 0.007), and CA19-9 (HR = 2.060, 95% CI = 1.192–3.562; *p* = 0.010) as independent OS risk factors in ICC (Table 2). Multivariate analysis identified IGF2BP2 (HR = 1.946, 95% CI = 1.024–3.698; *p* = 0.042), Ki-67 index (HR = 2.279, 95% CI = 1.090–4.765; *p* = 0.029), and CA19-9 (HR = 3.004, 95% CI = 1.512–5.970; *p* = 0.005) as independent RFS risk factors in ICC (Table 3).

## 4. Discussion

IGF2BP2 has emerged as a pan-cancer prognostic biomarker, with its overexpression consistently associated with poorer survival across glioma (including low-grade), HNSCC, LSCC, OSCC, LUAD, ESCC, PDAC, PC, HCC, and CRC [21,22,23,24,25,26,27,28,29,30,31,32,33]. Among the IGF2BPs, IGF2BP2 exhibits the strongest clinical association, promoting proliferation, invasion, metastasis, and an immunosuppressive microenvironment. Despite this oncogenic profile, its role in ICC remained undefined. Our integrated bioinformatic and tissue-level validation now addresses this gap.

In our ICC cohort, IGF2BP2 was significantly upregulated in tumors compared to adjacent bile duct tissues (*p* < 0.001), with 52.2% classified as having high expression. This pattern persisted across large-duct and small-duct subtypes (both *p* < 0.001). Expression also varied by histological subtype (*p* < 0.05), being higher in large-duct than in small-duct ICC, suggesting its utility as a subtyping biomarker complementary to existing panels (e.g., CK7/CK19/S100P for LD; CRP/N-cadherin for SD). While IGF2BP2 was correlated with the overall TNM stage (*p* = 0.030), the distribution across stages was non-linear. High expression was prominently enriched in Stage III (32.5% vs. 16.8% in the low-expression group), which was in line with its significant associations with vascular (*p* = 0.037) and nerve invasion (*p* = 0.011). Conversely, the frequency of Stage IV was lower in the high-expression group (5.1% vs. 11.2%). This represents a study limitation, likely reflecting the small Stage IV sample (*N* = 18; 8.0% of the cohort), which may introduce stochastic variation. Biologically, this disparity suggests that IGF2BP2 might primarily drive regional advancement and local invasiveness rather than distant metastasis. Larger multi-center cohorts are required to clarify these stage-specific dynamics. IGF2BP2 further correlated with serum markers (ALT, AST, ALP, GGT, CRP, creatinine, CA19-9), supporting combined detection for diagnosis/monitoring, though tissue-based assessment is more technically demanding than serum testing. Although IGF2BP2 is primarily an intracellular protein and there is currently a lack of direct evidence of its secretion into the bloodstream, its expression is correlated with established serum markers such as CA19-9 and AKP. Future studies could hypothesize that IGF2BP2-related signaling might be detectable via exosomal RNA or circulating tumor RNA (ctRNA), which offers a potential non-invasive window into the tumor’s molecular landscape.

Prognostically, elevated IGF2BP2 is associated with worse outcomes—for example, reduced survival in HNSCC (HR = 1.014, 95% CI = 1.004–1.025) and lower 1-year survival in PDAC (*p* = 0.017) [23,29]. In ICC, high IGF2BP2 predicts significantly shorter 5-year OS and RFS (*p* < 0.05), supporting its role as an independent prognostic factor. However, the limitations of a single-center cohort (sample size, potential follow-up gaps, and unelucidated mechanisms) warrant further study. Multivariate analyses also demonstrated that high IGF2BP2 expression was an independent prognostic factor for both OS (HR = 1.683, 95% CI = 1.015–2.791; *p* = 0.044) and RFS (HR = 1.946, 95% CI = 1.024–3.698; *p* = 0.042) in patients with ICC. The prognostic robustness of IGF2BP2 was consistently observed across different survival endpoints. However, we acknowledge that the retrospective nature of variable selection may introduce potential model instability. These findings warrant further validation in independent, prospective cohorts. Although tumor diameter >5 cm (HR = 2.155, 95% CI = 1.292–3.595; *p* = 0.003) is a well-established indicator of poor prognosis, our study demonstrates that IGF2BP2, as an independent biological marker, offers prognostic value that transcends the limitations of anatomical volume alone. This suggests that IGF2BP2 may drive malignant progression in ICC primarily by promoting immune evasion rather than merely enhancing cell proliferation and tumor growth.

The recent insights from Li et al. emphasize that IGF2BP2 functions not merely as an isolated stabilizer of specific mRNAs but as a central hub in post-transcriptional regulation [34]. As an oncogenic regulator, IGF2BP2 orchestrates the complex interplay between post-transcriptional modifications and the global transcriptional network. This implies that IGF2BP2 may act as a molecular bridge. Through the recognition of m^6^A sites, it not only enhances the stability of immune-related transcripts such as PD-L1 (CD274) but also modulates the expression of key transcription factors, thereby establishing a self-reinforcing immunosuppressive loop. Such integrative regulatory capacity may explain why IGF2BP2 overexpression serves as such a robust independent prognostic factor. It coordinates multi-layered gene expression programs that collectively foster an aggressive and immunologically cold tumor microenvironment. ICC employs the PD-1/PD-L1 checkpoint axis to evade immune surveillance [35,36,37], where PD-L1 binding inhibits T-cell signaling (PI3K-AKT-mTOR/Ras-MEK-ERK), inducing metabolic reprogramming and T-cell exhaustion to establish an immunosuppressive niche [14]. Clinically, PD-L1-high ICC exhibits aggressive features and reduced survival. The identification of reliable biomarkers is paramount for personalizing immunotherapy in intrahepatic cholangiocarcinoma (ICC). Recent bioinformatics-driven frameworks have increasingly demonstrated the power of immune-related molecular signatures in predicting both clinical outcomes and therapeutic responses. For instance, integrated genomic and transcriptomic profiling has identified specific gene sets that characterize the ‘immune-cold’ phenotype and predict resistance to checkpoint blockade [38]. Our findings align with this paradigm. IGF2BP2 overexpression not only predicts poor survival but also correlates with a suppressive tumor microenvironment defined by high PD-L1 expression and deficient CD8^+^ T-cell infiltration. Predictive analysis based on the GEPIA2 database revealed a significant positive correlation between IGF2BP2 expression levels and immune checkpoint genes in cholangiocarcinoma (correlation coefficient *R* > 0.3, *p* < 0.05), suggesting a potential association between this molecule and tumor immune evasion. While IGF2BP2-PD-L1 regulation in ICC remained unelucidated, our validation revealed the following: PD-L1 heterogeneity in tumor/immune cells; positive IGF2BP2-PD-L1 correlations (TPS: *R* = 0.215, *p* = 0.016; CPS: *R* = 0.295, *p* = 0.008); significantly elevated PD-L1 in IGF2BP2-high group (*p* < 0.05). Although the correlation coefficients between IGF2BP2 and PD-L1 ranged from 0.215 to 0.295, indicating weak to moderate strength, the statistically significant *p*-values (*p* < 0.05), along with evidence of spatial co-localization, underscore their potential biological relevance within the immune microenvironment of ICC. However, a limitation of this study regarding CD274 mRNA stability and expression levels should be noted. Constrained by the use of TMAs constructed from formalin-fixed paraffin-embedded (FFPE) tissue samples, our current analysis was confined to IHC for protein-level detection. Consequently, large-scale investigations of mRNA stability, such as actinomycin D chase assays or reverse transcription quantitative polymerase chain reaction (RT-qPCR) analyses, could not be performed within this cohort. Triple IHC/multiplex immunofluorescence concurrently associated high IGF2BP2 with PD-1^+^ cells and indicated reduced CD8^+^ T-cell infiltration. We acknowledge that the use of TMAs presents inherent challenges regarding intra-tumoral heterogeneity. Although we employed duplicate 1.5 mm cores for each case to enhance representativeness, this sampling method may not fully capture the entire landscape of PD-L1 heterogeneity or the full spectrum of immune infiltration variability across the whole tumor mass. Future studies utilizing whole-slide imaging or spatial transcriptomics will be instrumental in further validating these spatial relationships in a more comprehensive manner. Ideally, high infiltration of CD8^+^ TILs is indicative of enhanced antitumor immunity, while elevated Treg infiltration is associated with immune evasion [15]. Based on the positive correlation between IGF2BP2 and Tregs observed in TCGA-CHOL, we hypothesize that IGF2BP2 may not only suppress CD8^+^ T cell activity via the PD-L1/PD-1 axis but also contribute to maintaining the immune evasion status in ICC by recruiting or inducing Treg infiltration. Given that the tissue microarray (TMA) samples were exhausted after multiple rounds of staining and technical tissue loss occurred during the experimental process, it was difficult to conduct high-quality supplementary experiments on the original samples to validate our hypotheses. Although high IGF2BP2 expression was noted to be associated with the infiltration of CD4^+^ and FoxP3^+^ cells, the multiplex fluorescence staining yielded suboptimal visualization due to spatial overlap between FoxP3 (nuclear expression) and CD4 (membranous expression). To address this, we used additional clinical tissue sections from the corresponding cases and performed separate staining to clearly illustrate the expression distribution of IGF2BP2, CD4, and FoxP3.

We acknowledge that the current study is limited to clinicopathological correlations. In the absence of direct mechanistic evidence or functional data on checkpoint blockade response in ICC models, our proposed therapeutic strategy remains a hypothesis that requires future experimental verification. IGF2BP2 could serve as a core component of future multi-gene molecular signatures used to stratify patients for anti-PD-1/PD-L1 therapies, facilitating more precise clinical decision-making in ICC. However, the clinical implementation of PD-1/PD-L1 blockade in ICC is not without challenges, particularly regarding the safety profile. While these therapies aim to revitalize the exhausted immune microenvironment, they can also trigger a spectrum of immune-related adverse events (irAEs). Specifically, hepatobiliary toxicities represent a significant concern in patients with underlying biliary malignancies. Recent evidence from He et al. highlights the intricate nature of immune-related hepatobiliary injuries, emphasizing the need for vigilant monitoring and personalized management during checkpoint inhibition [39]. Integrating the targeting of IGF2BP2 with PD-1/PD-L1 inhibitors may offer a synergistic anti-tumor effect. However, the potential for exacerbated hepatic inflammation or biliary complications must be carefully evaluated in future clinical trials to ensure a favorable therapeutic window.

## 5. Conclusions

In conclusion, IGF2BP2 is overexpressed in intrahepatic cholangiocarcinoma (ICC) tumors and acts as an independent prognostic risk factor for reduced overall survival (OS) and recurrence-free survival (RFS). Its expression correlates with key clinicopathological features, including histological type, vascular invasion, nerve invasion, and TNM stage, as well as serum indices such as alkaline phosphatase (AKP) and carbohydrate antigen 19-9 (CA19-9). Furthermore, clinical observations suggest that high IGF2BP2 expression may be potentially associated with immune evasion, which is characterized by increased PD-L1/PD-1 expression and reduced CD8^+^ T cell infiltration. The combined targeting of IGF2BP2 and PD-L1/PD-1 blockade may represent a potential therapeutic strategy for ICC, and this warrants further validation through functional and clinical intervention studies.

## Figures and Tables

**Table 1:** Clinicopathological Characteristics and Serum Indices of 224 ICC patients.

Clinical Characteristics	Total (N = 224)	IGF2BP2-High (N = 117)	IGF2BP2-Low (N = 107)	*p*-Value
Age				0.329
<65	137	68 (58.1%)	69 (64.5%)	
≥65	87	49 (41.9%)	38 (35.5%)	
Gender				0.271
Male	117	57 (48.7%)	60 (56.1%)	
Female	107	60 (51.3%)	47 (43.9%)	
Tumor number				0.699
Single	184	95 (81.2%)	89 (83.2%)	
Multiple	40	22 (18.8%)	18 (16.8%)	
Maximum diameter of tumor				0.084
≤5 cm	114	66 (56.4%)	48 (44.9%)	
>5 cm	110	51 (43.6%)	59 (55.1%)	
Histologic type				<**0.001**
LD	69	48 (41.0%)	21 (19.6%)	
SD	155	69 (59.0%)	86 (80.4%)	
Vascular invasion				**0.037**
No	168	81 (69.2%)	87 (81.3%)	
Yes	56	36 (30.8%)	20 (18.7%)	
Nerve invasion				**0.011**
No	131	59 (50.4%)	72 (67.3%)	
Yes	93	58 (49.6%)	35 (32.7%)	
TNM				**0.030**
Stage I	103	49 (41.9%)	54 (50.5%)	
Stage II	47	24 (20.5%)	23 (21.5%)	
Stage III	56	38 (32.5%)	18 (16.8%)	
Stage IV	18	6 (5.1%)	12 (11.2%)	
Tumor differentiation degree				0.312
Poorly	40	18 (15.4%)	22 (20.6%)	
Moderately/Well	184	99 (84.6%)	85 (79.4%)	
Ki67				0.183
≤20%	78	36 (30.8%)	42 (39.3%)	
>20%	146	81 (69.2%)	65 (60.7%)	
ALT (U/L)				**0.021**
Normal: 5~40	178	86 (73.5%)	92 (86.0%)	
Abnormal: ≥40	46	31 (26.5%)	15 (14.0%)	
AST (U/L)				**0.016**
Normal: 4~40	187	91 (77.8%)	96 (89.7%)	
Abnormal: ≥40	37	26 (22.2%)	11 (10.3%)	
AKP (U/L)				**0.015**
Normal: 40~150	170	81 (69.2%)	89 (83.2%)	
Abnormal: ≥150	54	36 (30.8%)	18 (16.8%)	
GGT (U/L)				**0.029**
Normal: <50	92	40 (34.2%)	52 (48.6%)	
Abnormal: ≥50	132	77 (65.8%)	55 (51.4%)	
Alb (g/L)				0.077
Normal: 35~55	206	104 (88.9%)	102 (95.3%)	
Abnormal: <35	18	13 (11.1%)	5 (4.7%)	
CRP (mg/L)				**0.044**
Normal: <10	164	79 (67.5%)	85 (79.4%)	
Abnormal: ≥10	60	38 (32.5%)	22 (20.6%)	
Cr (umol/L)				**0.037**
Normal: 44~133	207	104 (88.9%)	103 (96.3%)	
Abnormal: <44	17	13 (11.1%)	4 (3.7%)	
AFP (ng/mL)				0.250
Normal: <25	214	110 (94.0%)	104 (97.2%)	
Abnormal: ≥25	10	7 (6.0%)	3 (2.8%)	
CEA (ng/mL)				0.527
Normal: <5	189	97 (82.9%)	92 (86.0%)	
Abnormal: ≥5	35	20 (17.1%)	15 (14.0%)	
CA19-9 (U/mL)				**0.006**
Abnormal: <5	20	13 (11.1%)	7 (6.5%)	
Normal: 5~37	77	28 (23.9%)	49 (45.8%)	
Abnormal: 37~1000	82	47 (40.2%)	35 (32.7%)	
Abnormal: >1000	45	29 (24.8%)	16 (15.0%)	

**Note:** Bold indicates *p* < 0.05. **Abbreviations:** TNM, tumor-node-metastasis; ALT, alanine aminotransferase; AST, aspartate aminotransferase; AKP, alkaline phosphatase; GGT, gamma-glutamyl transpeptidase; Alb, albumin; CRP, C-reactive protein; Cr, creatinine; AFP, alpha fetoprotein; CEA, carcinoembryonic antigen; CA19-9, carbohydrate antigen 19-9.

**Table 2:** Factors associated with OS of ICC patients: univariate and multivariate analysis (*n* = 224).

Clinical Characteristics	Univariate Analysis		Multivariate Analysis	
	*p*	HR (95% CI)	*p*	HR (95% CI)
The expression of IGF2BP2 (High vs. Low)	0.017	1.747 (1.104~2.765)	**0.044**	1.683 (1.015~2.791)
Age (≥65 vs. <65)	0.104	1.449 (0.926~2.268)		
Gender (Female vs. Male)	0.087	1.482 (0.944~2.325)		
Tumor number (Multiple vs. Single)	0.044	1.743 (1.014~2.995)	0.316	1.395 (0.728~2.676)
Maximum diameter of tumor (>5 cm vs. ≤5 cm)	0.035	1.633 (1.034~2.577)	**0.003**	2.155 (1.292~3.595)
Histologic type (LD vs. SD)	0.042	1.624 (1.018~2.591)	0.976	0.992 (0.582~1.691)
TNM (Stages II–IV vs. Stage I)	<0.001	2.736 (1.666~4.492)	0.615	1.273 (0.496~3.267)
Tumor differentiation degree (Well vs. Poorly)	0.624	0.865 (0.484~1.546)		
Vascular invasion (Yes vs. No)	0.001	2.217 (1.39~3.535)	0.234	1.423 (0.796~2.546)
Nerve invasion (Yes vs. No)	0.033	1.628 (1.04~2.548)	0.195	1.398 (0.842~2.32)
Ki67 (>20% vs. ≤20%)	0.228	1.347 (0.83~2.186)		
ALT (Abnormal vs. Normal)	0.27	1.34 (0.797~2.253)		
AST (Abnormal vs. Normal)	0.026	1.824 (1.075~3.095)	0.42	0.752 (0.376~1.504)
AKP (Abnormal vs. Normal)	<0.001	2.423 (1.515~3.873)	0.061	1.819 (0.972~3.401)
GGT (Abnormal vs. Normal)	0.003	2.157 (1.305~3.564)	0.579	1.192 (0.641~2.219)
Alb (Abnormal vs. Normal)	<0.001	3.388 (1.82~6.304)	**0.007**	2.653 (1.304~5.396)
Cr (Abnormal vs. Normal)	0.711	1.171 (0.508~2.701)		
CRP (Abnormal vs. Normal)	0.126	1.461 (0.899~2.374)		
AFP (Abnormal vs. Normal)	0.161	1.914 (0.772~4.75)		
CEA (Abnormal vs. Normal)	0.076	1.671 (0.948~2.946)		
CA19-9 (Abnormal vs. Normal)	<0.001	2.858 (1.713~4.768)	**0.01**	2.06 (1.192~3.562)

**Note:** Bold indicates *p* < 0.05; HR, hazard ratio; CI, confidence interval. **Abbreviations:** TNM, tumor-node-metastasis; ALT, alanine aminotransferase; AST, aspartate aminotransferase; AKP, alkaline phosphatase; GGT, gamma-glutamyl transpeptidase; Alb, albumin; CRP, C-reactive protein; Cr, creatinine; AFP, alpha fetoprotein; CEA, carcinoembryonic antigen; CA19-9, carbohydrate antigen 19-9.

**Table 3:** Factors Associated with RFS of ICC Patients: Univariate and Multivariate Analysis (*n* = 142).

Clinical Characteristics	Univariate Analysis		Multivariate Analysis	
	*p*	HR (95% CI)	*p*	HR (95% CI)
The expression of IGF2BP2 (High vs. Low)	0.047	1.852 (1.007~3.404)	**0.042**	1.946 (1.024~3.698)
Age (≥65 vs. <65)	0.932	1.027 (0.559~1.885)		
Gender (Female vs. Male)	0.079	1.707 (0.94~3.101)		
Tumor number (Multiple vs. Single)	0.112	1.817 (0.87~3.798)		
Maximum diameter of tumor (>5 cm vs. ≤5 cm)	0.036	1.906 (1.044~3.48)	0.053	0.729 (0.53~1.004)
Histologic type (LD vs. SD)	0.533	1.231 (0.641~2.365)		
TNM (Stages II–IV vs. Stage I)	0.092	1.692 (0.918~3.117)		
Tumor differentiation degree (Well vs. Poorly)	0.040	0.52 (0.279~0.971)	0.057	0.525 (0.27~1.019)
Vascular invasion (Yes vs. No)	0.023	2.043 (1.102~3.785)	0.502	0.879 (0.603~1.282)
Nerve invasion (Yes vs. No)	0.095	1.655 (0.915~2.994)		
Ki67 (>20% vs. ≤20%)	0.032	2.16 (1.066~4.374)	**0.029**	2.279 (1.09~4.765)
ALT (Abnormal vs. Normal)	0.346	1.423 (0.683~2.964)		
AST (Abnormal vs. Normal)	0.084	1.916 (0.915~4.008)		
AKP (Abnormal vs. Normal)	0.081	1.899 (0.924~3.902)		
GGT (Abnormal vs. Normal)	0.338	1.342 (0.735~2.451)		
Alb (Abnormal vs. Normal)	0.233	2.047 (0.631~6.639)		
Cr (Abnormal vs. Normal)	0.359	1.626 (0.576~4.59)		
CRP (Abnormal vs. Normal)	0.204	1.523 (0.795~2.916)		
AFP (Abnormal vs. Normal)	0.792	1.21 (0.292~5.012)		
CEA (Abnormal vs. Normal)	0.061	1.97 (0.97~4)		
CA19-9 (Abnormal vs. Normal)	0.002	3.004 (1.512~5.97)	**0.005**	2.802 (1.366~5.747)

**Note:** Bold indicates *p* < 0.05; HR, hazard ratio; CI, confidence interval. **Abbreviations:** TNM, tumor-node-metastasis; ALT, alanine aminotransferase; AST, aspartate aminotransferase; AKP, alkaline phosphatase; GGT, gamma-glutamyl transpeptidase; Alb, albumin; CRP, C-reactive protein; Cr, creatinine; AFP, alpha fetoprotein; CEA, carcinoembryonic antigen; CA19-9, carbohydrate antigen 19-9.

## Data Availability

The raw data supporting the conclusions of this article will be made available by the authors on request.

## Abbreviations

The following abbreviations are used in this manuscript:

TME	Tumor microenvironment
ICC	Intrahepatic cholangiocarcinoma
IGF2BP2	Insulin-like growth factor 2 mRNA-binding protein 2
PD-L1	Programmed death-ligand 1
PD-1	Programmed cell death protein1
IHC	Immunohistochemistry
TMA	Tissue microarray
OS	Overall survival
RFS	Recurrence-free survival
CCA/CHOL	Cholangiocarcinoma
m^6^A	N^6^-methyladenosine
LD	Large-duct
SD	Small-duct
TNM	Tumor-node-metastasis
ALT	Alanine aminotransferase
AST	Aspartate aminotransferase
AKP	Alkaline phosphatase
GGT	Gamma-glutamyl transpeptidase
Alb	Albumin
CRP	C-reactive protein
Cr	Creatinine
AFP	Alpha fetoprotein
CEA	Carcinoembryonic antigen
CA19-9	Carbohydrate antigen 19-9
GEO	Gene Expression Omnibus
TCGA	The Cancer Genome Atlas
TISIDB	Tumor Immune System Interaction Database
